# Hierarchical WS_2_-WO_3_ Nanohybrids with Flower-like p-n Heterostructures for Trimethylamine Detection

**DOI:** 10.3390/nano14161322

**Published:** 2024-08-06

**Authors:** Dan Meng, Shunjiang Ran, Lei Zhang, Xiaoguang San, Yue Zhang, Yu Zheng, Jian Qi

**Affiliations:** 1 College of Chemical Engineering, Shenyang University of Chemical Technology, Shenyang 110142, China; mengdan@syuct.edu.cn (D.M.); z2022004@stu.syuct.edu.cn (S.R.); laylazhang@syuct.edu.cn (L.Z.); 2020048@stu.syuct.edu.cn (Y.Z.); 2111030219@stu.syuct.edu.cn (Y.Z.); 2 State Key Laboratory of Biochemical Engineering, Institute of Process Engineering, Chinese Academy of Sciences, Beijing 100190, China

**Keywords:** WS_2_/WO_3_, p-n heterostructures, trimethylamine sensing, flower-like hierarchical structure

## Abstract

The detection of trimethylamine (TMA) is critically important due to its toxic and flammable nature, which poses significant risks to human health and the environment. However, achieving high response, rapid kinetics, selectivity, and low operating temperatures in TMA sensing remains challenging. In this study, WS_2_/WO_3_ nanohybrids with flower-like hierarchical structures were synthesized via an in situ sulfurization process, utilizing varying amounts of thioacetamide to control the sulfurization state of WO_3_. These novel hierarchical WS_2_/WO_3_ nanohybrids exhibit remarkable selectivity towards TMA, as well as rapid response and recovery characteristics. Specially, the optimal WS_2_/WO_3_ sensor, composed of 5% WS_2_/WO_3_ nanohybrids, demonstrates exceptional TMA sensing performance, including a high response (19.45 at 10 ppm), good repeatability, reliable long-term stability, and a low theoretical detection limit (15.96 ppb). The superior sensing capabilities of the WS_2_/WO_3_ nanohybrids are attributed to the formation of p-n heterojunctions at the interface, the unique hierarchical structures, and the catalytic activity of WS_2_. Overall, this work provides a straightforward and versatile approach for synthesizing multifunctional nanomaterials by combining metal oxide micro-flowers with transition metal dichalcogenide nanoflakes for applications in monitoring TMA in complex environments.

## 1. Introduction

Trimethylamine (TMA) is one of the volatile organic compounds (VOCs) and is a colorless, flammable gas with a strong and fishy odor. It is naturally produced during the decomposition of organic matter and is commonly found in various biological processes [1]. TMA can serve as an essential intermediate in industrial chemical synthesis and is used to produce various products, including pharmaceuticals, pesticides, and surfactants [2]. Inhalation of TMA vapors can cause health issues such as coughing, upper respiratory system irritation, breathing difficulties, and lung edema [3]. The presence and concentration of TMA are often monitored as indicators of spoilage in food products, particularly fish and seafood [4,5]. Additionally, TMA has been detected in human exhaled breath at concentrations ranging from 0.1 to 0.2 ppm, suggesting its potential as a biomarker for renal diseases [6]. Therefore, monitoring TMA levels can provide valuable insights into the freshness and quality of seafood products, early disease diagnosis, and environmental monitoring.

In recent years, metal oxide semiconductors such as WO_3_ [7], SnO_2_ [8,9,10], ZnO [11,12], NiO [13,14], and In_2_O_3_ [15,16] have been extensively utilized as sensing materials for detecting various gases in the environment due to their relatively low cost, ease of integration, and broad gas detection capabilities [17,18]. Among these, WO_3_ stands out due to its unique properties, including a wide bandgap and excellent catalytic activity, making it particularly effective for detecting a range of gases [19,20]. Notably, the sensing ability of WO_3_-based sensors is affected by operating temperature [21], often showing high sensor signals and fast response times at relatively high temperatures (200–300 °C) due to chemical kinetics considerations. Furthermore, advancements in nanotechnology have significantly enhanced the performance of WO_3_ sensors. Strategies such as constructing p-n or n-n heterojunctions, doping with noble metals, and regulating oxygen vacancies have been explored to improve WO_3_’s sensing performance [22,23]. The construction of p-n or n-n heterojunctions is particularly notable for its simplicity and effectiveness. Tungsten disulfide (WS_2_), a representative W-based transition metal dichalcogenide (TMD), has garnered significant attention as a platform for heterostructure sensing applications due to its graphene-like structure, appropriate bandgap, high surface activity, ease of surface functionalization, and high electron mobility [24,25]. Recent studies have reported the construction of WS_2_/WO_3_ heterojunctions for toxic gas detection. For instance, nanoflower-like WS_2_/WO_3_ composites have shown superior response to NO_2_ at room temperature [26]. Additionally, the heterostructured nanohybrids of WS_2_/WO_3_ exhibit better acetone sensing properties compared to pristine WS_2_ or WO_3_, making it suitable for noninvasive diagnosis of diseases such as diabetes and lung cancer [27]. Inspired by these findings, it is proposed that constructing WS_2_/WO_3_ heterojunctions on WO_3_ micro-nanostructures is a promising strategy to enhance TMA sensing performance. However, there is currently a lack of research on the TMA sensing performance of WS_2_/WO_3_ composites. Moreover, achieving well-defined WS_2_/WO_3_ composites with consistent micro-nanostructures remains a significant challenge. Further investigation is necessary to understand the sensing mechanism of WS_2_/WO_3_ composites, which holds great relevance in this field.

In this work, WO_3_ with a flower-like hierarchical structure was synthesized using a facile solvothermal method. An in situ sulfurization process was conducted with varying amounts of thioacetamide to control the sulfurization state of WO_3_, resulting in the formation of WS_2_/WO_3_ nanohybrids. The TMA gas sensing performance of the WS_2_/WO_3_ sensors was systematically investigated. As expected, the TMA sensing performance of WS_2_/WO_3_ showed a significant enhancement compared to that of pure WO_3_. Notably, the sensor based on 5%-WS_2_/WO_3_ (optimized thioacetamide content) demonstrated superior sensing response towards TMA, a low detection limit, high selectivity, good stability, and repeatability, positioning it as a promising candidate for practical TMA sensor applications.

## 2. Experimental Section

### 2.1. Materials and Chemicals

Chemicals in the experiment including tungstic acid (H_2_WO_4_), hydrogen peroxide (H_2_O_2_), acetonitrile (CH_3_CN), hydrochloric acid (HCl), oxalic acid (C_2_H_2_O_4_·2H_2_O), urea (H_2_NCONH_2_), thioacetamide (C_2_H_5_NS), trimethylamine (C_3_H_9_N), formaldehyde (HCHO), ethanol (CH_3_CH_2_OH), acetone (CH_3_COCH_3_), methanol (CH_3_OH), and ammonia (NH_3_) are analytical grade reagents, negating the necessity for additional purification prior to experimentation.

### 2.2. Synthesis of WS_2_/WO_3_ Nanohybrids with Flower-like Hierarchical Structures

**Figure 1:**
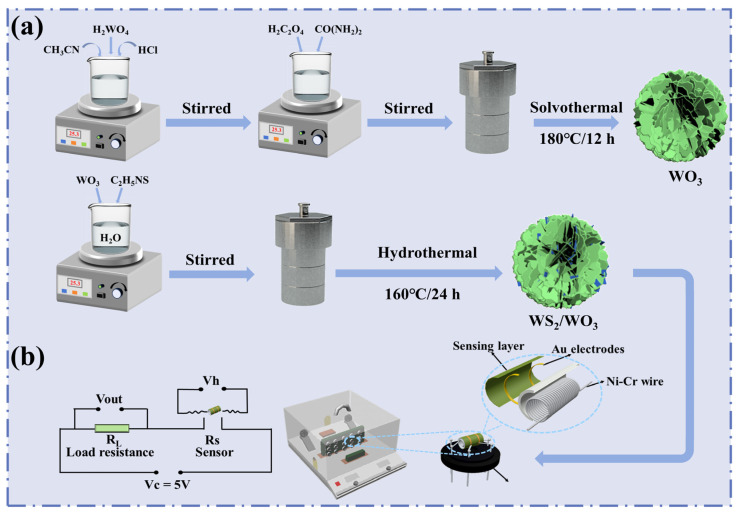
Schematic illustrations of (**a**) the synthesis process of WS_2_/WO_3_ nanohybrids, and (**b**) the gas sensing measurement system.

The synthesis process of WS_2_/WO_3_ nanohybrids with flower-like hierarchical structures is schematically illustrated in Figure 1a. Initially, 2.5 g of H_2_WO_4_ was dispersed in a solvent mixture composed of 50 mL deionized water and 34 mL (30 wt%) H_2_O_2_. The solution was stirred at 95 °C in a water bath until the H_2_WO_4_ completely dissolved. After cooling, deionized water was added to dilute the solution to 200 mL, resulting in an H_2_WO_4_ precursor solution with a concentration of 0.05 M. Subsequently, 0.05 g C_2_H_2_O_4_·2H_2_O and 0.05 g urea were sequentially added to the solvent mixture, which consisted of 7.5 mL of the H_2_WO_4_ precursor solution, 31.5 mL CH_3_CN, and 2.5 mL HCl (3 M), and stirred for 30 min. The solution was then transferred to a 50 mL Teflon-lined stainless steel autoclave and reacted at 180 °C for 12 h. Upon cooling to room temperature, the precipitate was subjected to six cycles of ethanol washing and subsequently dried overnight at 60 °C, resulting in WO_3_ with flower-like hierarchical structures.

To obtain WS_2_/WO_3_ flower-like hierarchical structures, the prepared WO_3_ (115 mg) was dispersed in 30 mL of deionized water and stirred for 1 h. The obtained dispersion was then mixed with appropriate amounts of C_2_H_5_NS, corresponding to [S]/[W] molar ratios of 0.03, 0.05, 0.07, and 0.09, respectively, and magnetically stirred for an additional hour. The mixed solution was transferred to a 50 mL Teflon-lined stainless steel autoclave and maintained at 160 °C for 24 h. After the reaction, the precipitates were collected, sequentially washed with deionized water and ethanol, and dried in a vacuum drying oven at 60 °C to yield the WS_2_/WO_3_ composites. The resulting WS_2_/WO_3_ composites, synthesized with [S]/[W] molar ratios of 0.03, 0.05, 0.07, and 0.09, were designated as 3%-WS_2_/WO_3_, 5%-WS_2_/WO_3_, 7%-WS_2_/WO_3_, and 9%-WS_2_/WO_3_, respectively.

### 2.3. Characterization

The crystalline structures of WS_2_/WO_3_ were analyzed using X-ray diffraction (XRD, Shimadzu XRD-6100) with Cu Kα radiation. The morphology of the products was examined by scanning electron microscopy (SEM, ZEISS Ultra Plus, ZEISS, Oberkochen, Germany), which was equipped with energy-dispersive X-ray spectroscopy (EDS) to obtain elemental distribution maps. Further detailed characterization of the microstructures was performed using high-resolution transmission electron microscopy (HRTEM) on a transmission electron microscopy system (TEM, JEOL, JEM-2100F, Tokyo, Japan). The charge carrier transport properties of the products were analyzed by electrochemical impedance spectroscopy (EIS) using an electrochemical workstation (Shanghai Chenhua Science Technology Corp., Ltd., Shanghai, China) with a standard three-electrode configuration. A glassy carbon electrode was employed as the working electrode, while a graphite rod and a saturated Hg/HgCl_2_ electrode served as the counter and reference electrodes, respectively. To prepare the working electrode, the glassy carbon electrode was initially polished with an alumina suspension. Subsequently, 2 mg of the WS_2_-WO_3_ nanohybrids was dispersed in a solution containing 97 μL of deionized water, 100 μL of ethanol, and 3 μL of Nafion solution. The resulting mixture underwent ultrasonic treatment for 30 min to achieve a homogeneous ink. Finally, 50 μL of the ink was dropped onto the surface of the glassy carbon electrode and allowed to dry at room temperature. The electrochemical tests were performed in a 0.5 M Na_2_SO_4_ solution (pH~7) at room temperature, with a frequency range spanning from 0.1 Hz to 10,000 Hz.

### 2.4. Fabrication and Measurement of Sensors

The fabrication process of the gas sensor was conducted as follows: Initially, an appropriate quantity of either WS_2_/WO_3_ or pure WO_3_ powder was mixed with ethanol to produce a homogeneous slurry. This slurry was then coated onto the external surface of an Al_2_O_3_ ceramic tube equipped with two Au electrodes and four Pt wires at both ends, forming a thick sensing film. A Ni-Cr alloy wire, serving as a heater to control the working temperature, was passed through the ceramic tube and welded onto a hexagonal base along with the Pt wires, thus forming the sensor (Figure 1b). Finally, the gas sensor was placed on a TS60 desktop (Winsen Electronics Co., Ltd., Zhengzhou, China) and subjected to aging at 400 °C for 48 h to ensure stability. Gas-sensitive measurements of the prepared sensors were performed under static conditions using a commercial WS-30A system (Winsen Electronics Co., Ltd., Zhengzhou, China). The schematic structure of the gas sensor and the test circuit diagram are depicted in Figure 1b. In this study, a constant loop voltage (V_c_) of 5 V was maintained throughout the testing process. The operational principle of the test circuit is governed by the following parameters: R_L_ represents the load resistance, V_h_ denotes the heating voltage, and V_out_ signifies the output load voltage. The ratio of R_a_ (resistance in the air) to R_g_ (resistance in the target gas) is defined as the response.

## 3. Results and Discussion

### 3.1. Structural and Morphological Characteristics

**Figure 2:**
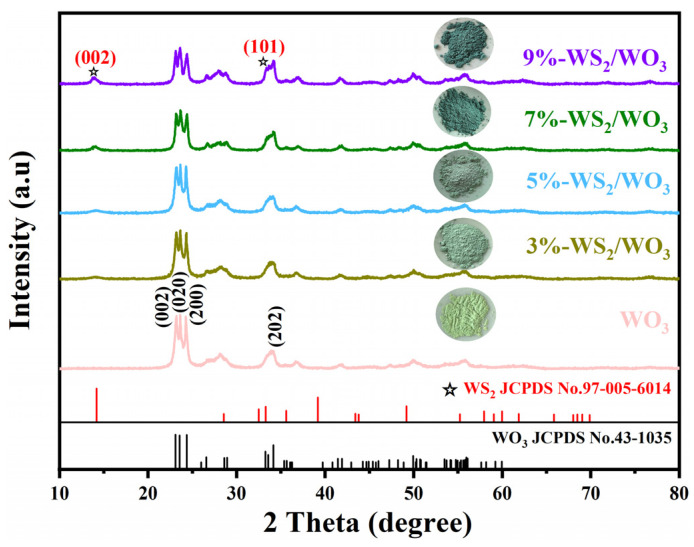
XRD patterns of WO_3_ and WS_2_/WO_3_ nanohybrids.

Figure 2 presents the XRD spectra of the samples. It is evident that all XRD patterns of the WO_3_ sample exhibit sharp diffraction peaks without any impurity peaks, indicating that the synthesized WO_3_ possesses high purity and good crystallinity. The diffraction peaks at 2θ = 23.12°, 23.59°, 24.38°, and 34.16° correspond to the (002), (020), (200), and (202) crystal planes of the monoclinic structure of WO_3_ (JCPDS No. 43-1035) [28]. Following the sulfurization of WO_3_, diffraction peaks at 2θ = 14.16° and 33.29°, corresponding to the (002) and (101) crystal planes of WS_2_ (JCPDS No. 97-005-6014) [29], are observed, confirming the formation of WS_2_/WO_3_ composites. The intensity of the WS_2_ diffraction peaks increases with the increase in the added amount of C_2_H_5_NS, suggesting that more WS_2_ is being deposited on the WO_3_ surface. In addition, the color of WO_3_ products is gradually changed into dark green with the increase in the added amount of C_2_H_5_NS, which also indicates the increased sulfurization degree of WO_3_.

**Figure 3:**
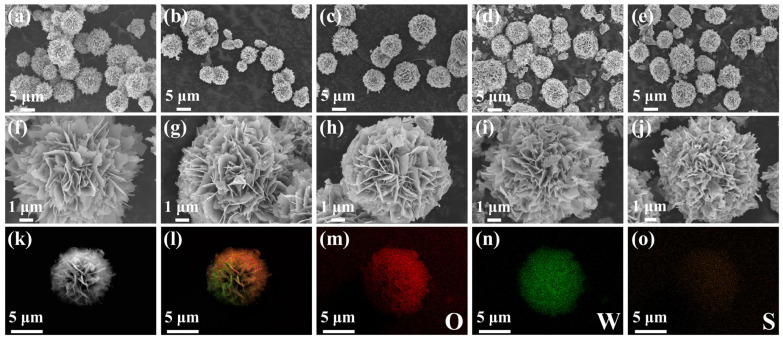
SEM images of (**a**,**f**) WO_3_, (**b**,**g**) 3%-WS_2_/WO_3_, (**c**,**h**) 5%-WS_2_/WO_3_, (**d**,**i**) 7%-WS_2_/WO_3_, and (**e**,**j**) 9%-WS_2_/WO_3_; (**k**–**o**) EDS mapping images of O, W, and S elements on the 5%-WS_2_/WO_3_ nanohybrids.

From the SEM images of WO_3_ (Figure 3a), it is evident that pure WO_3_ shows well-defined flower-like structures with relatively uniform sizes of approximately 4 to 5 μm. Further observation (Figure 3f) reveals that these flower-like structures are composed of many loosely connected nanosheets with a thickness of approximately 10 to 30 nm, which are nearly perpendicular to a central point. This arrangement creates multiple voids between the nanosheets, leading to an increased active surface area and enhanced sensing capability. After in situ sulfurization, the WS_2_/WO_3_ nanohybrids retain the flower-like structures, as shown in Figure 3b–e. Additionally, tiny nanoflakes begin to appear on the surface of WO_3_ micro-flowers. With the increase in the added amount of C_2_H_5_NS, the distribution of nanosheet-assembled WO_3_ micro-flowers becomes denser due to the intensified sulfurization degree of WO_3_ and the WS_2_ tiny nanoflakes can be well observed (Figure 3h–j). Figure 3k–o presents the EDS element mapping of 5%-WS_2_/WO_3_, which demonstrates the uniform distribution of O, W, and S elements on the surface. This observation confirms the presence of both WO_3_ and WS_2_ in the samples.

**Figure 4:**
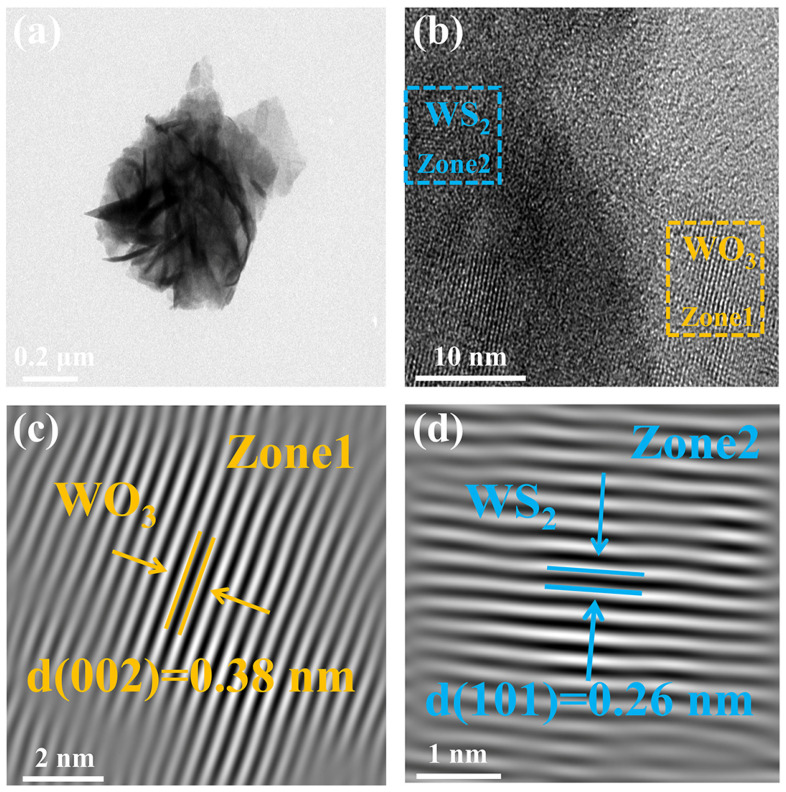
(**a**) TEM and (**b**–**d**) HRTEM images of 5%-WS_2_/WO_3_ nanohybrids.

Detailed microstructural analysis of the 5%-WS_2_/WO_3_ nanohybrids was conducted using TEM and HRTEM. As shown in Figure 4a, a flower-like structure composed of numerous loosely connected thin nanosheets is observed, consistent with the FE-SEM images. HRTEM images in Figure 4b–d reveal the finer structure and interfaces within the 5%-WS_2_/WO_3_ nanohybrids. The lattice fringes at distances of 0.38 nm and 0.26 nm correspond to the (002) crystal plane of WO_3_ and the (101) crystal plane of WS_2_, respectively. These observations underscore the distinct crystallization characteristics of the two materials. Specifically, the HRTEM images provide compelling evidence of the formation of WS_2_/WO_3_ heterojunctions, which facilitates interfacial electron transfer and consequently enhances the sensing ability.

### 3.2. Gas Sensing Properties

**Figure 5:**
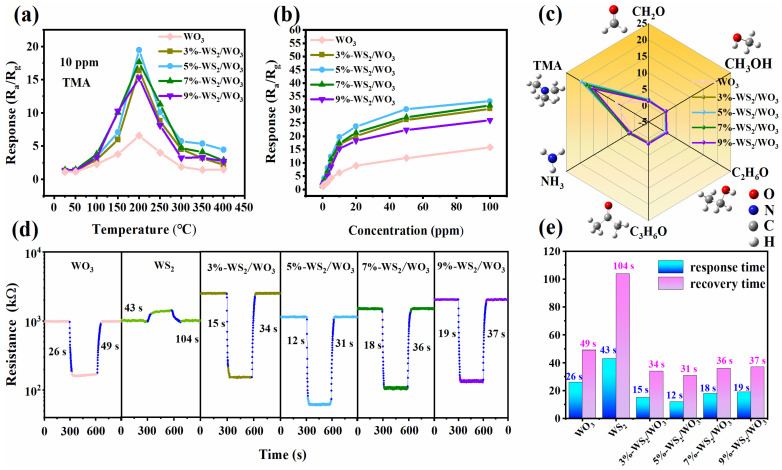
The sensing performance of WO_3_ and WS_2_/WO_3_ sensors: (**a**) response to 10 ppm TMA at 25–400 °C; (**b**) response with TMA concentration (0.1–100 ppm) at 200 °C; (**c**) selectivity to 10 ppm different target gases at 200 °C; (**d**) dynamic response–recovery curves to 10 ppm TMA (the blue line in each curve represents the response and recovery time interval) and (**e**) corresponding response and recovery time at 200 °C.

Many factors influence the performance of gas sensors, with operating temperature being particularly significant due to its impact on the adsorption–desorption reaction kinetics of gas molecules on the semiconductor surface [30]. To determine the optimal operating temperature, the responses of WO_3_, 3%-WS_2_/WO_3_, 5%-WS_2_/WO_3_, 7%-WS_2_/WO_3_, and 9%-WS_2_/WO_3_ sensors to 10 ppm TMA were evaluated over a temperature range of 25–400 °C. As illustrated in Figure 5a, the response values of all sensors increased progressively with rising temperature, peaking at 200 °C, after which the response values declined with further temperature increments. Thus, the optimal temperature for all sensors is identified as 200 °C, yielding response values of 6.58, 16.40, 19.45, 17.67, and 15.29 for WO_3_, 3%-WS_2_/WO_3_, 5%-WS_2_/WO_3_, 7%-WS_2_/WO_3_, and 9%-WS_2_/WO_3_, respectively. Clearly, the introduction of WS_2_ enhances the sensing response at lower temperature ranges, thereby improving the sensors’ reliability and stability for practical applications.

The sensor responses to various TMA concentrations (0.1–100 ppm) are presented in Figure 5b. As the TMA concentration increases from 1 to 10 ppm, all sensors exhibit a rapid response increase. Beyond this range, the response increment slows progressively with higher TMA concentrations, likely due to adsorption saturation of surface active sites [31]. Additionally, the 5%-WS_2_/WO_3_ sensor consistently exhibits the highest response at each TMA concentration and is capable of detecting TMA at concentrations as low as 0.1 ppm, highlighting the sensitization effect of WS_2_/WO_3_. Figure 5c illustrates the responses of sensors to 10 ppm of various interfering gases (TMA, ethanol, methanol, formaldehyde, acetone, and ammonia) at the optimal temperature of 200 °C. It is evident that the response of all sensors to TMA is significantly higher than to other gases, indicating good selectivity for TMA. Notably, the 5%-WS_2_/WO_3_ sensor shows the highest response to TMA, suggesting its potential application in detecting TMA in complex gas environments.

Figure 5d illustrates the response–recovery curves of the WO_3_, WS_2_, and WS_2_/WO_3_ sensors. Upon exposure to TMA, the WO_3_ and WS_2_/WO_3_ sensors exhibit typical n-type semiconductor characteristics, evidenced by a decrease in resistance when exposed to the reducing gas (TMA). Conversely, the WS_2_ sensor demonstrates a reversed trend in resistance change upon exposure to TMA, indicative of a typical p-type semiconductor characteristic, where the resistance increases in the presence of a reducing gas (TMA). After the removal of TMA gas, all sensors revert to their baseline levels, underscoring their effective response and recovery capacities. The time taken to achieve 90% of the total resistance change during adsorption and desorption processes is termed the response and recovery times, respectively. As shown in Figure 5e, the WS_2_/WO_3_ sensor exhibits shorter response and recovery times compared to the WO_3_ sensor, implying that partial sulfurization-functionalized WO_3_ enhances the response–recovery characteristics. These findings indicate that the 5%-WS_2_/WO_3_ sensor possesses excellent TMA sensing capabilities, which was further investigated at its optimum operating temperature of 200 °C.

**Figure 6:**
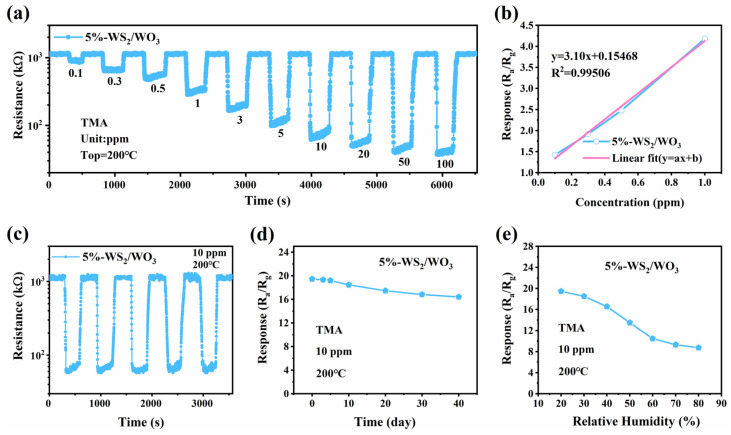
The sensing performance of the 5%-WS_2_/WO_3_ sensor at 200 °C: (**a**) dynamic response–recovery curves to different TMA concentrations; (**b**) experimental response signal (blue line) and linear fitted response (pink line) as a function of TMA concentration; (**c**) eepeatability curves toward 10 ppm TMA; (**d**) response to TMA over 40 days; and (**e**) response to 10 ppm TMA under different relative humidity conditions.

The transient response and recovery curves of the 5%-WS_2_/WO_3_ sensor to different TMA concentrations at 200 °C (0.1–100 ppm) are shown in Figure 6a. When the TMA gas is injected, the resistance decreases rapidly and stabilizes after a short period. Upon removal of TMA from the chamber, the resistance quickly returns to its initial value. This stable and repeatable sensing response behavior, even at lower concentrations (0.1 ppm), suggests its suitability for detecting TMA gas across a wide concentration range. Additionally, the response versus the TMA concentration (0.1–1 ppm) shows a good linear relationship, with a fitting slope of 3.10 ppm^−1^ (Figure 6b). The theoretical detection limit is calculated to be approximately 15.96 ppb based on the signal-to-noise ratio, indicating its potential application in monitoring ppb-level TMA in the environment.

The repeatability of the 5%-WS_2_/WO_3_ sensor was investigated by exposing it to 10 ppm TMA in five cycles of response–recovery at 200 °C. As shown in Figure 6c, each cycle shows similar response and recovery trends, and the initial resistance value shows only slight fluctuations in each trial, implying good repeatability. Moreover, the TMA sensing response of the 5%-WS_2_/WO_3_ sensor was measured for 40 days to assess long-term stability. As shown in Figure 6d, although the response values change during the testing process, they degrade only slightly, indicating good stability of the sensor, which is favorable for practical applications. The effect of humidity on gas sensing response was investigated, as depicted in Figure 6e. The response of the 5%-WS_2_/WO_3_ sensor to 10 ppm TMA exhibits a slight decrease within the relative humidity (RH) range of 20% to 40%, while it exhibits an obvious decrease at RH levels exceeding 50%. This reduction can be attributed to the competition between surface oxygen and the OH group of H_2_O for adsorption and reaction sites with TMA molecules [32]. Notably, even at a high RH level of 80%, the sensor maintains a response of 8.73 for 10 ppm TMA, implying impressive sensing capability under high-humidity conditions. The operating temperature, response time, and minimum detection limit of various trimethylamine sensors are summarized in Table 1. It can be seen that the 5%-WS_2_/WO_3_ sensor has ideal trimethylamine detection sensing characteristics.

### 3.3. Gas Sensing Mechanism

**Figure 7:**
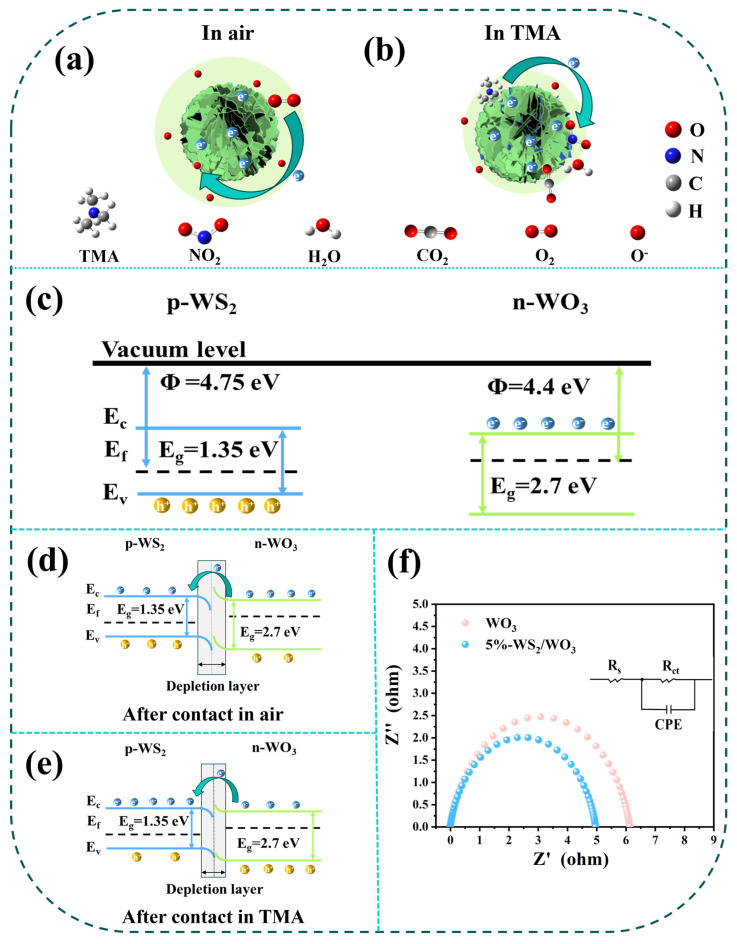
(**a**,**b**) Schematic diagram of the gas sensing mechanism of the WS_2_/WO_3_ sensor; (**c**–**e**) schematic of energy bands of WS_2_/WO_3_ under different conditions; (**f**) the Nyquist plots of WO_3_ and 5%-WS_2_/WO_3_ were measured in the frequency range of 0.1~10,000 Hz.

In general, metal oxide sensors operate based on the principle that their electrical conductivity changes in the presence of a target gas. In this study, WO_3_ and WS_2_/WO_3_ sensors exhibit typical n-type sensing characteristics, where electrons are the primary charge carriers in the sensing process. In the air atmosphere, oxygen molecules adsorb onto the surface of the material, capture electrons from the conduction band, and convert into negative oxygen ions (O_2_^−^, O^−^, or O^2−^). Consequently, the number of carriers in the material decreases, forming an electron depletion layer on the surface, which hinders electron transfer and increases the sensor’s resistance (Figure 7a). When the sensor is exposed to TMA, a reaction occurs between the TMA molecules and the adsorbed oxygen molecules. During this process, the electrons captured by the oxygen molecules are released back to the conduction band, thinning the electron depletion layer and thereby reducing the sensor’s resistance in the TMA atmosphere (Figure 7b). The reaction equation is as follows [43,44]:

(1) $$O_{2}\left(gas\right)\to O_{2}\left(ads\right)$$

(2) $$O_{2}\left(ads\right)+e^{-}\to O_{2}^{-}\left(ads\right)\left(T\leq 100\;℃\right)$$

(3) $$O_{2}\left(ads\right)+e^{-}\to 2O^{-}(ads)(100\;℃\leq T\leq 300\;℃)$$

(4) $$2{\left({CH}_{3}\right)}_{3}N+{43O}^{-}\left(ads\right)\to {2NO}_{2}+{12CO}_{2}+15H_{2}O+{43e}^{-}$$

As shown in Figure 7c–e, due to the higher Fermi level of the WO_3_ semiconductor, electrons flow from WO_3_ to p-type WS_2_, while holes flow from WS_2_ to WO_3_ until the Fermi level achieves equilibrium [45]. The WO_3_ surface is an electron depletion region, and the WS_2_ surface is a negative charge region. Due to the built-in field, the p-n heterojunction leads to the barrier of electron conduction [46]. Upon exposure to TMA, the heterojunctions between WO_3_ and WS_2_ create additional resistance modulation by altering the potential barriers and the two electron depletion layers. Consequently, the WS_2_/WO_3_ heterojunction configuration significantly enhances the sensor’s response ability. Secondly, the sensor’s performance benefits from the unique flower-like structures, which promote the penetration of oxygen and TMA molecules into the sensing layers, facilitating rapid gas diffusion. Furthermore, the formation of p-n heterojunctions results in the creation of a transition layer, “S-W-O”, at the interface [47], which effectively accumulates additional free electrons and facilitates charge transfer. The EIS measurement results (Figure 7f) confirm the enhanced transfer and migration of charge carriers through an internal electric field and interfacial interaction at the heterojunction of WS_2_/WO_3_ composites. This increases the carrier density at the interface, generating more adsorbed oxygen and allowing more TMA to participate in the reaction. This results in excellent gas sensing performance. For these comprehensive reasons, the WS_2_/WO_3_ sensor exhibits superior sensing performance towards TMA.

## 4. Conclusions

In summary, WS_2_/WO_3_ nanohybrids with flower-like hierarchical structures were synthesized via an in situ sulfurization process, employing varying amounts of thioacetamide to control the sulfurization state of WO_3_. The synthesized WS_2_/WO_3_ nanohybrids exhibited significantly enhanced TMA sensing performance compared to pure WO_3_ micro-flowers. The optimal WS_2_/WO_3_ sensor, comprising 5% WS_2_/WO_3_ nanohybrids, demonstrated superior sensing response towards TMA, low detection limits, high selectivity, good repeatability, and reliable long-term stability. The enhanced sensing performance of the WS_2_/WO_3_ nanohybrids is attributed to the formation of p-n heterojunctions at the interface, the unique hierarchical structures, and the catalytic activity of WS_2_. This work offers new insights into the preparation of multifunctional nanomaterials by combining metal oxide micro-flowers with TMD nanoflakes, which hold potential for monitoring TMA in complex atmospheres.

## Figures and Tables

**Table 1:** Comparison of gas sensing properties with other reported sensors to TMA.

Materials	Temperature (°C)	Concentration (ppm)	Response (R_a_/R_g_)	Res./Rec. Time (s)	LOD (ppm)	References
Au@Pt/α-Fe_2_O_3_	150	100	32	5/74	1	[33]
Rh/ZnO	180	10	11.3	93/110	1	[34]
ZFMI	RT	10	6.23	132/43	5	[35]
TiO_2_-NiFe_2_O_4_	307	10	12	50/45	0.1	[36]
Au-WO_3_	268	100	41.56	1/323	1	[37]
Co_3_O_4_/SnO_2_	170	5	9.3	19/29	1	[38]
TiO_2_/Ti_2_O(PO_4_)_2_	170	100	87.46	14.6/630	0.2	[39]
α-Fe_2_O_3_/α-MoO_3_	80	24	18.6	12/106	10	[40]
In-WO_3_	115	50	7.36	11/40	1	[41]
Co_3_O_4_/In_2_O_3_	200	10	11.67	25/68	1	[42]
WS_2_/WO_3_	200	10	19.45	12/31	0.1	This work

## Data Availability

Data are contained within the article.
